# Supplementary material for: Wildfires increasingly impact western US fluvial networks
Source: Nat Commun. 2021 Apr 30;12:2484. doi: 10.1038/s41467-021-22747-3 (PMC8087797; doi:10.1038/s41467-021-22747-3)
Supplement: Supplementary file 1 — Supplementary Information [file 41467_2021_22747_MOESM1_ESM.pdf]

Supplementary information for **Wildfires increasingly impact western US fluvial networks**

Grady Ball[1]*, Peter Regier[2]*†, Ricardo González-Pinzón[2], Justin Reale[3], David Van Horn[4]

*These authors contributed equally to this study
†now at Pacific Northwest National Laboratory

Corresponding authors: David Van Horn vanhorn@unm.edu & Ricardo González-Pinzón gonzaric@unm.edu

Affiliations:
1. Water Resources Graduate Program, University of New Mexico, Albuquerque, NM, USA
2. Department of Civil, Construction & Environmental Engineering, University of New Mexico, Albuquerque, NM, USA
3. U.S. Army Corps of Engineers, Albuquerque District, Albuquerque, NM, USA
4. Department of Biology, University of New Mexico, Albuquerque, NM, USA

# Supplementary Figures

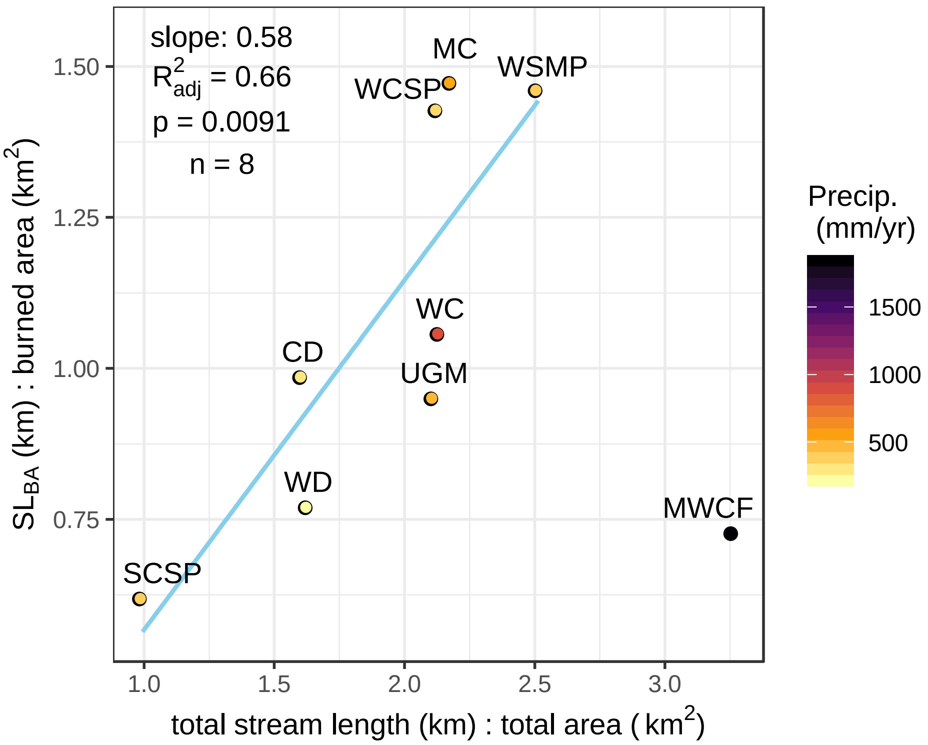


**Supplementary Figure 1.** Correlation between total stream length:total area and $SL_{BA}$:burned area ratios by ecoregion. Marine West Coast Forest is plotted, but excluded from regression statistics. Each ecoregion is colored by mean annual precipitation (mm/yr). See Figure 1 and Table S1 for ecoregion abbreviations.

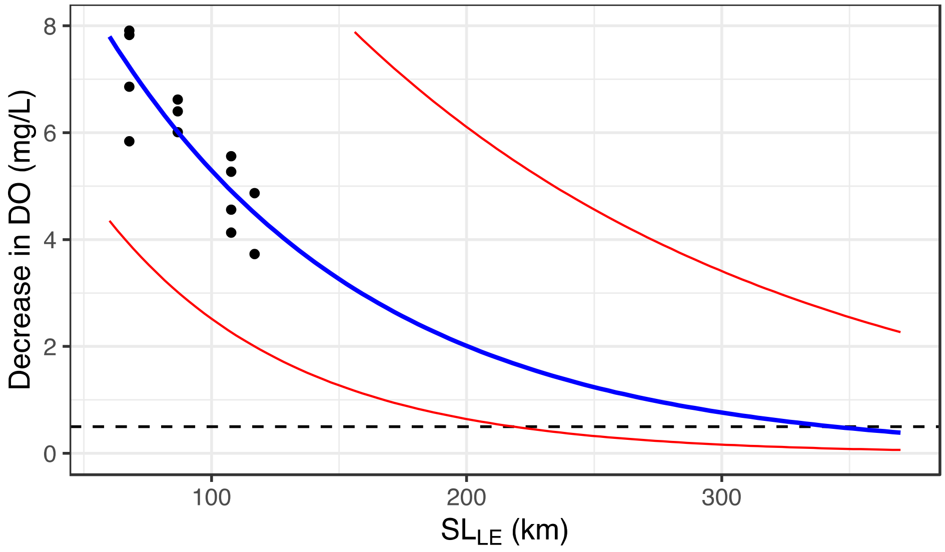


**Supplementary Figure 2.** Exponential decay model fitted to dissolved oxygen sags from four storm events in 2011 following the Las Conchas wildfire. Each storm event included had high-quality data for at least three of the four sites. The 95% confidence interval is represented by red fit lines representing the upper and lower limits. Additional details are presented in the Methods section of the manuscript.

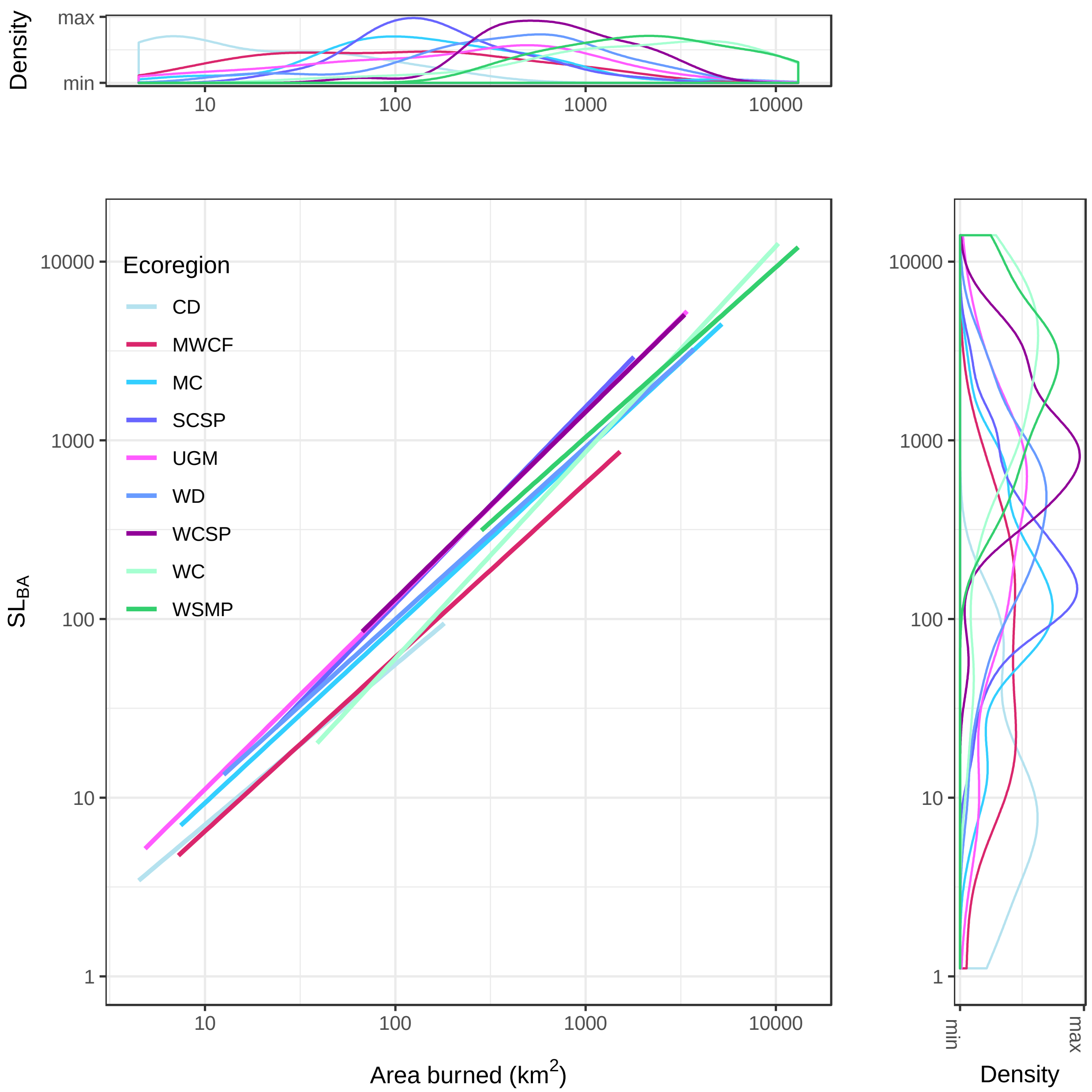


**Supplementary Figure 3.** Regressions between annual sums of $SL_{BA}$ and area burned by ecoregion. Density plots along each axis show the distribution of data for each variable (see Table S3). See Figure 1 and Table S1 for ecoregion abbreviations.

# Supplementary Tables

**Supplementary Table 1.** Fire statistics by ecoregion.

| | | Fires | | Area burned | | | Stream length affected | | |
|---|---|---|---|---|---|---|---|---|---|
| Ecoregion | Abbreviation | Number of fires | Fires per year | Total area ($km^2$) | Area burned ($km^2$) | Percent area burned | Total length (km) | $SL_{BA}$ (km) | Percent length affected |
| Cold Deserts | CD | 2683 | 87 | 1006646 | 107527 | 10.7% | 1620148 | 106310 | 6.6% |
| Marine West Coast Forest | MWCF | 31 | 1 | 86182 | 476 | 0.6% | 281372 | 348 | 0.1% |
| Mediterranean California | MC | 933 | 30 | 165005 | 30161 | 18.3% | 360102 | 44511 | 12.4% |
| South Central Semiarid Prairies | SCSP | 266 | 9 | 233927 | 7088 | 3.0% | 232602 | 4409 | 1.9% |
| Upper Gila Mountains | UGM | 542 | 17 | 108628 | 21586 | 19.9% | 229562 | 20584 | 9.0% |
| Warm Deserts | WD | 425 | 14 | 317115 | 11950 | 3.8% | 517029 | 9237 | 1.8% |
| West-Central Semiarid Prairies | WCSP | 375 | 12 | 297725 | 13545 | 4.5% | 633736 | 19375 | 3.1% |
| Western Cordillera | WC | 2147 | 69 | 814204 | 101832 | 12.5% | 1739608 | 107963 | 6.2% |
| Western Sierra Madre Piedmont | WSMP | 275 | 9 | 42936 | 7751 | 18.1% | 107908 | 11343 | 10.5% |
| Total/Average | | 7677 | 28 | 3072368 | 301917 | 10.1% | 5722067 | 324080 | 5.7% |

**Supplementary Table 2.** Annual sums for $SL_{BA}$ presented in Figure 2B. See Figure 1 and Table S1 for ecoregion abbreviations.

| Year | Annual $SL_{BA}$ (km / year) by ecoregion | | | | | | | | | |
|---|---|---|---|---|---|---|---|---|---|---|
| | CD | MWCF | MC | SCSP | UGM | WD | WCSP | WC | WSMP | Sum |
| 1984 | 2014 | 0 | 990 | 0 | 11 | 83 | 737 | 196 | 0 | 4031 |
| 1985 | 8847 | 5 | 3051 | 6 | 78 | 26 | 13 | 664 | 48 | 12737 |
| 1986 | 4095 | 0 | 772 | 21 | 42 | 16 | 9 | 1387 | 127 | 6470 |
| 1987 | 1559 | 90 | 379 | 14 | 177 | 76 | 0 | 5396 | 194 | 7885 |
| 1988 | 1190 | 27 | 830 | 73 | 151 | 155 | 978 | 6960 | 194 | 10559 |
| 1989 | 692 | 0 | 1197 | 139 | 155 | 278 | 4 | 1248 | 584 | 4297 |
| 1990 | 858 | 14 | 1360 | 16 | 313 | 56 | 143 | 857 | 125 | 3743 |
| 1991 | 415 | 0 | 58 | 1 | 31 | 12 | 1028 | 302 | 118 | 1965 |
| 1992 | 1220 | 0 | 361 | 13 | 77 | 119 | 82 | 1626 | 0 | 3499 |
| 1993 | 262 | 0 | 2122 | 18 | 311 | 829 | 0 | 15 | 558 | 4115 |
| 1994 | 3095 | 0 | 881 | 180 | 567 | 866 | 412 | 3780 | 1175 | 10956 |
| 1995 | 2870 | 0 | 454 | 64 | 720 | 691 | 82 | 45 | 152 | 5077 |
| 1996 | 10110 | 8 | 3384 | 244 | 642 | 401 | 1514 | 3663 | 18 | 19984 |
| 1997 | 474 | 0 | 1113 | 63 | 132 | 84 | 11 | 53 | 138 | 2069 |
| 1998 | 1880 | 1 | 733 | 189 | 143 | 191 | 141 | 561 | 108 | 3948 |
| 1999 | 12181 | 15 | 1992 | 53 | 280 | 287 | 872 | 2644 | 364 | 18687 |
| 2000 | 4943 | 0 | 480 | 207 | 578 | 160 | 492 | 7260 | 268 | 14388 |
| 2001 | 2589 | 0 | 271 | 25 | 260 | 47 | 148 | 2422 | 275 | 6037 |
| 2002 | 2015 | 0 | 1241 | 218 | 2667 | 12 | 616 | 9736 | 1078 | 17584 |
| 2003 | 344 | 75 | 4996 | 8 | 837 | 252 | 1480 | 4152 | 225 | 12371 |
| 2004 | 572 | 0 | 814 | 0 | 1236 | 65 | 74 | 814 | 148 | 3723 |
| 2005 | 3243 | 0 | 580 | 48 | 2582 | 2364 | 214 | 2146 | 85 | 11262 |
| 2006 | 4196 | 4 | 3010 | 593 | 553 | 739 | 3626 | 4279 | 340 | 17341 |
| 2007 | 7081 | 0 | 4410 | 11 | 249 | 119 | 491 | 10178 | 35 | 22574 |
| 2008 | 1176 | 93 | 3632 | 498 | 363 | 94 | 691 | 5961 | 471 | 12979 |
| 2009 | 792 | 9 | 2093 | 509 | 799 | 118 | 64 | 1306 | 1173 | 6863 |
| 2010 | 3314 | 2 | 368 | 22 | 394 | 86 | 586 | 851 | 134 | 5755 |
| 2011 | 4327 | 6 | 447 | 1019 | 3029 | 614 | 670 | 2255 | 2803 | 15170 |
| 2012 | 14046 | 0 | 899 | 158 | 1591 | 151 | 4028 | 12993 | 247 | 34112 |
| 2013 | 2096 | 0 | 954 | 0 | 885 | 237 | 0 | 4304 | 72 | 8549 |
| 2014 | 3813 | 0 | 640 | 0 | 732 | 6 | 170 | 9907 | 85 | 15353 |
| min | 262 | 0 | 58 | 0 | 11 | 6 | 0 | 15 | 0 | |
| max | 14046 | 93 | 4996 | 1019 | 3029 | 2364 | 4028 | 12993 | 2803 | |
| average | 3429 | 11 | 1436 | 142 | 664 | 298 | 625 | 3483 | 366 | 10454 |

**Supplementary Table 3.** Regression statistics for Figure 2. See Figure 1 and Table S1 for ecoregion abbreviations.

| Biome | $R^2_{adj}$ | slope ($km/km^2$) | p-value | n |
|---|---|---|---|---|
| CD | 0.82 | 0.92 | p<0.0001 | 31 |
| MWCF | 0.54 | 0.52 | 0.0025 | 13 |
| MC | 0.96 | 1.49 | p<0.0001 | 31 |
| SCSP | 0.88 | 0.56 | p<0.0001 | 27 |
| UGM | 0.95 | 0.94 | p<0.0001 | 31 |
| WD | 0.88 | 0.47 | p<0.0001 | 31 |
| WCSP | 0.91 | 1.35 | p<0.0001 | 28 |
| WC | 0.84 | 1.02 | p<0.0001 | 31 |
| WSMP | 0.97 | 1.59 | p<0.0001 | 29 |
| All | 0.91 | 0.98 | p<0.0001 | 252 |
